# Functional expression of calcium‐permeable canonical transient receptor potential 4‐containing channels promotes migration of medulloblastoma cells

**DOI:** 10.1113/JP274659

**Published:** 2017-07-20

**Authors:** Wei‐Chun Wei, Wan‐Chen Huang, Yu‐Ping Lin, Esther B. E. Becker, Olaf Ansorge, Veit Flockerzi, Daniele Conti, Giovanna Cenacchi, Maike D. Glitsch

**Affiliations:** ^1^ Department of Physiology, Anatomy and Genetics University of Oxford Oxford OX1 3PT UK; ^2^ Institute of Cellular and Organismic Biology Academia Sinica Taipei 115 Taiwan; ^3^ Nuffield Department of Clinical Neurosciences University of Oxford Oxford OX3 9DU UK; ^4^ Experimental and Clinical Pharmacology and Toxicology Saarland University Homburg Germany; ^5^ Department of Biomedical and Neuromotor Science University of Bologna Italy

**Keywords:** cerebellum, medulloblastoma, OGR1, proton sensing G protein coupled receptors, Transient Receptor Potential Channels, TRPC4

## Abstract

**Key points:**

- The proton sensing ovarian cancer G protein coupled receptor 1 (OGR1, aka GPR68) promotes expression of the canonical transient receptor potential channel subunit TRPC4 in normal and transformed cerebellar granule precursor (DAOY) cells.
- OGR1 and TRPC4 are prominently expressed in healthy cerebellar tissue throughout postnatal development and in primary cerebellar medulloblastoma tissues.
- Activation of TRPC4‐containing channels in DAOY cells, but not non‐transformed granule precursor cells, results in prominent increases in [Ca^2+^]_i_ and promotes cell motility in wound healing and transwell migration assays.
- Medulloblastoma cells not arising from granule precursor cells show neither prominent rises in [Ca^2+^]_i_ nor enhanced motility in response to TRPC4 activation unless they overexpressTRPC4.
- Our results suggest that OGR1 enhances expression of TRPC4‐containing channels that contribute to enhanced invasion and metastasis of granule precursor‐derived human medulloblastoma.

**Abstract:**

Aberrant intracellular Ca^2+^ signalling contributes to the formation and progression of a range of distinct pathologies including cancers. Rises in intracellular Ca^2+^ concentration occur in response to Ca^2+^ influx through plasma membrane channels and Ca^2+^ release from intracellular Ca^2+^ stores, which can be mobilized in response to activation of cell surface receptors. Ovarian cancer G protein coupled receptor 1 (OGR1, aka GPR68) is a proton‐sensing G_q_‐coupled receptor that is most highly expressed in cerebellum. Medulloblastoma (MB) is the most common paediatric brain tumour that arises from cerebellar precursor cells. We found that nine distinct human MB samples all expressed OGR1. In both normal granule cells and the transformed human cerebellar granule cell line DAOY, OGR1 promoted expression of the proton‐potentiated member of the canonical transient receptor potential (TRPC) channel family, TRPC4. Consistent with a role for TRPC4 in MB, we found that all MB samples also expressed TRPC4. In DAOY cells, activation of TRPC4‐containing channels resulted in large Ca^2+^ influx and enhanced migration, while in normal cerebellar granule (precursor) cells and MB cells not derived from granule precursors, only small levels of Ca^2+^ influx and no enhanced migration were observed. Our results suggest that OGR1‐dependent increases in TRPC4 expression may favour formation of highly Ca^2+^‐permeable TRPC4‐containing channels that promote transformed granule cell migration. Increased motility of cancer cells is a prerequisite for cancer invasion and metastasis, and our findings may point towards a key role for TRPC4 in progression of certain types of MB.

## Key points

- The proton sensing ovarian cancer G protein coupled receptor 1 (OGR1, aka GPR68) promotes expression of the canonical transient receptor potential channel subunit TRPC4 in normal and transformed cerebellar granule precursor (DAOY) cells.
- OGR1 and TRPC4 are prominently expressed in healthy cerebellar tissue throughout postnatal development and in primary cerebellar medulloblastoma tissues.
- Activation of TRPC4‐containing channels in DAOY cells, but not non‐transformed granule precursor cells, results in prominent increases in [Ca^2+^]_i_ and promotes cell motility in wound healing and transwell migration assays.
- Medulloblastoma cells not arising from granule precursor cells show neither prominent rises in [Ca^2+^]_i_ nor enhanced motility in response to TRPC4 activation unless they overexpressTRPC4.
- Our results suggest that OGR1 enhances expression of TRPC4‐containing channels that contribute to enhanced invasion and metastasis of granule precursor‐derived human medulloblastoma.

**Abbreviations**
CaSR: extracellular Ca^2+^ sensing receptor
(−)EA: (−)englerin A
ERK: extracellular signal regulated kinase
GPCR: G protein coupled receptor
GPR: G protein receptor
MB: medulloblastoma
OGR1: ovarian cancer G protein coupled receptor 1
SHH: sonic hedgehog
TDAG8: T cell death associated gene 8
TRPC: canonical transient receptor potential
WNT: wingless/integrated

## Introduction

Aberrant Ca^2+^ signalling contributes to cancer formation and progression (Monteith *et al*. 2007, 2012; Chen *et al*. 2013; Cross *et al*. 2014; Stewart *et al*. 2015). Ca^2+^ influx through plasma membrane ion channels and Ca^2+^ release from stores both contribute to intracellular Ca^2+^ signals, and many different types of ion channels have been shown to play key roles in various aspects of cancer formation and progression, including enhanced motility and facilitation of metastatic spread (Matteo, 2007; Arcangeli *et al*. 2009; Becchetti & Arcangeli, 2010; Cuddapah & Sontheimer, 2011; Pla *et al*. 2012; Fiorio Pla & Munaron, 2014; Lang & Stournaras, 2014; Nielsen *et al*. 2014; Pardo & Stuhmer, 2014; Schwab & Stock, 2014; Turner & Sontheimer, 2014; Litan & Langhans, 2015; Stock & Schwab, 2015). The same is true for plasma membrane receptors linking to Ca^2+^ release from intracellular Ca^2+^ stores (Bergner & Huber, 2008; Theman & Collins, 2009; Wypych & Pomorski, 2013), which can further contribute to intracellular Ca^2+^ signalling by activating or inhibiting ion channels.

One consequence of transformation is an alteration of cell metabolism, which contributes to acidification of the interstitial fluid of solid tumours. Intriguingly, this acidification promotes cancer formation and spread (Fais *et al*. 2014; Peppicelli *et al*. 2014; Gillies & Gatenby, 2015; Justus *et al*. 2015), though the exact mechanisms are not entirely clear. It is highly likely that proton‐sensing receptors and ion channels play a key role in sensing extracellular acidification of the interstitial tumour fluid, changes in which they then communicate to the cells in which they are expressed, thus influencing cellular processes and allowing cells to respond to the altered environment. Proton sensing receptors include the proton‐sensing G protein coupled receptors (GPCRs) OGR1 (aka GPR68), G protein receptor 4 (GPR4) (Ludwig *et al*. 2003) and T cell death associated gene 8 (TDAG8) (Wang *et al*. 2004), as well as a number of distinct ion channels (Holzer, 2009; Glitsch, 2011).

MB is the most common paediatric brain tumour and accounts for 12–25% of all childhood central nervous system tumours (Bartlett *et al*. 2013). Seven main phenotypes have been described, of which the classical and desmoplastic phenotype make up the vast majority of cases (80% and 15%, respectively) (Bartlett *et al*. 2013). More recently, genetic and transcriptome analyses have revealed the existence of at least four distinct subtypes based on the main signalling pathways affected: sonic hedgehog (SHH), wingless/integrated (WNT), group 3 (possibly Notch), and group 4 (possibly Myc) (Taylor *et al*. 2012; Bartlett *et al*. 2013; Coluccia *et al*. 2016). There is no straightforward correlation of phenotypes to genotypes, though it appears that desmoplastic MB belongs to the SHH group (Coluccia *et al*. 2016). Importantly, for desmoplastic MB, the cell of origin is well established. This MB subtype arises from granule precursor cells of the cerebellum (Kim *et al*. 2003; Pilkington, 2005) which populate the external granule cell layer and proliferate after birth. Once these precursors exit the cell cycle, they migrate through the molecular layer and populate the internal granule cell layer, where they complete the differentiation process (White & Sillitoe, 2013).

We have previously shown that, in the human desmoplastic MB cell line DAOY, Ca^2+^ release from intracellular Ca^2+^ stores following stimulation of OGR1 links to activation of ERK signalling, providing a mechanistic explanation of how extracellular acidification may impact gene transcription (Huang *et al*. 2008). Intriguingly, under differentiating conditions, OGR1‐dependent Ca^2+^ signalling and extracellular signal regulated kinase (ERK) activation were impaired, as was TRPC4 expression (Huang *et al*. 2009). This raises the intriguing possibility that OGR1 signalling and TRPC4 expression levels might be linked.

DAOY cells were derived from a desmoplastic MB, the cell of origin of which is the cerebellar granule precursor cells (Kim *et al*. 2003; Pilkington, 2005), and when addressing a role for OGR1 in primary cerebellar granule cells, we found that under physiological conditions, OGR1 can be under the inhibitory control of the extracellular Ca^2+^ sensing receptor, CaSR (Wei *et al*. 2015). This CaSR‐dependent regulation of OGR1 signalling was absent in DAOY cells despite CaSR being expressed (Wei *et al*. 2015), pointing towards a role for deregulation of OGR1 in transformed granule cells.

We wanted to address whether or not OGR1 signalling and TRPC4 expression were linked in primary granule cells and to establish a physiological role for TRPC4 in transformed and normal cerebellar cells in an attempt to elucidate events and processes leading and contributing to transformation.

## Methods

### Medulloblastoma cell line culture

DAOY, ONS76 and UW228‐1 cells were cultured in Dulbecco's modified Eagle's medium (DMEM) supplemented with 10% fetal calf serum (FCS) and 100 U ml^−1^ penicillin and streptomycin. Cells were cultured as described in Huang *et al*. (2008).

### Isolation of DAOY clones

DAOY cells were trypsinated at 70% confluency, spun down and resuspended in DMEM with all supplements. Cells were counted and plated in 96‐well plates at such dilutions that wells contained 10, 1 or 0.1 cells. Wells were monitored on a daily basis and those containing more than one cell clone were immediately dismissed. Successfully grown individual clones were transferred into increasingly larger well plates as they continued to expand and finally transferred into culturing flasks; this took between 4 and 8 weeks for the different clones. A total of eight clones were successfully isolated, which were all tested for TRPC4, TRPC5 and OGR1 expression levels using RT‐PCR and qPCR (see below) relative to wild‐type DAOY cells. Clones with the lowest TRPC4 and OGR1 expression (DAOY clones 6 and 3, respectively) were subsequently used for further experiments.

### Granule cell cultures

Cerebellar granule cells were established as described (Bilimoria & Bonni, 2008) at P6 and cultured for up to 15 days. In order to obtain cerebellar tissue, B57BL6 mice were killed by cervical dislocation. This procedure is listed as a Schedule 1 procedure (Appropriate methods of humane killing) under the Animals (Scientific Procedures) Act 1986 (UK) and did not require ethical permission as it was not a regulated procedure under the Act. As required by law, however, all personnel were trained and verified competent before carrying out the procedure. Mice were bred and housed in conditions in accordance with the Animals (Scientific Procedures) Act 1986. They were held under PPL number 30/3301 (service licence held by Ms Carol Williams).

Granule cells were grown in a 5% CO_2_ humidified atmosphere at 37°C in Gibco Basal Medium Eagle (Thermo Fisher Scientific, Waltham, MA, USA) supplemented with 10% FCS, 2 mm l‐glutamine, 4 unit ml^−1^ penicillin, 100 mg ml^−1^ streptomycin and 25 mm KCl. Cytosine 1‐α‐d‐arabinofuranoside (AraC, 10 μm, Sigma‐Aldrich, St Louis, MO, USA) was added at DIV1 to prevent further proliferation of cells, and 25 mm glucose was added at DIV3 and DIV8 without changing the medium. *Ogr1* knockout mice were a generous gift from Drs K. Seuwen and T. Suply (Novartis, Basel, Switzerland).

### Human medulloblastoma tissue

Snap frozen (*n* = 8) or fresh (*n* = 1) MB tissue surplus to diagnostic requirements were collected with approval by the local ethics committee (reference: 06/Q1605/141). Matching paraffin‐embedded samples were reviewed for confirmation of diagnosis and tumours were classified according to the WHO scheme for tumours of the nervous system (2007) by Dr Olaf Ansorge who also provided the samples. Table 1 lists the classification.

**Table 1:** Classification of tumours according to the WHO scheme for tumours of the nervous system (2007)

MB1	Nodular phenotype and tiny amount of healthy cerebellar tissue
MB2	Classic phenotype with no apparent differentiation
MB3	Strong differentiation (no further classification given)
MB4	Predominantly classic phenotype with very little differentiation
MB5	Classic phenotype with no apparent differentiation
MB6	Nodular phenotype
MB7	Anaplastic phenotype
MB8	Predominantly classic phenotype with very little differentiation
MB9	Classic phenotype

### Fluorescence imaging experiments

The intracellular Ca^2+^ signal was evaluated as previously described (Huang *et al*. 2008). All solutions were made using HPLC‐grade water. DAOY cells grown on coverslips were loaded with Fura‐2‐AM (4 μm, Molecular Probes/Thermo Fisher Scientific) in the standard external solution (145 mm NaCl, 10 mm Hepes, 2.8 mm KCl, 2 mm CaCl_2_, 2 mm MgCl_2_ and 10 mm glucose, pH 7.35) for 45 min at room temperature. Cells were then washed 3 times and left to recover for 15 min in standard external solution. Coverslips were then placed on a recording chamber, and submerged with standard external solution. For experiments under Ca^2+^‐free conditions, Ca^2+^ was omitted from the standard extermal solution, and 0.1 mm EGTA was added. Fluorescence ratios for Fura‐2 AM (usually 340 nm/380 nm) were measured every 2 s with a fluorometric system from Till Photonics (Martinsried, Germany). Either extracellular pH was dropped from pH 7.35 to pH 6.0 or drugs were added at indicated time (usually 1 min after recording started). In some experiments, cells were exposed to drugs prior to and during the imaging experiment. Control experiments with solvents were carried out on the same experimental days and preparations as all other experiments.

For imaging experiments in acute cerebellar slices, slices were incubated as described in Kirischuk & Verkhratsky (1996). Sagittal cerebellar slices were cut from P18 and P19 C57BL6 wild‐type mice, as previously described (Glitsch, 2010). Migrating granule cells in the molecular layer were identified by their elongated, spindle‐like cell body shape.

Fluorescence signals were analysed using Igor Pro 6.30 (WaveMetrics, Lake Oswego, OR, USA) and are represented as ratios of the fluorescence signals measured at 340 nm over 380 nm (Δ*F*), except for cerebellar slices in which fluorescence signals were measured at 356 nm over 380 nm. Average graphs were constructed by averaging all waves obtained for one experimental condition; control and test experiments were always performed on the same day.

### Evaluation of live cell numbers

DAOY cells (1 × 10^6^) were plated on 60 mm dishes in the absence and presence of 10 μm (−)englerin A ((−)EA) in culture medium with or without 10% FCS . After 12 h, cells were trypsinized and stained with 0.4% Gibco trypan‐blue (Thermo Fisher Scientific). Live, unstained cells were counted by using a haemocytometer.

### Proliferation assay

5‐Brom‐2′‐deoxy‐uridine (BrdU) Cell Proliferation Assay (Millipore, Billerica, MA, USA) was used for quantification of cell DNA synthesis and cellular proliferation according to manufacturer's instructions. Cells were plated in 96‐well plate and cultured with culture medium in the presence of BrdU labelling mixture and the agonist under investigation (AraC; Sigma‐Aldrich; 10 μm), pluronic acid (Life Technologies/Thermo Fisher Scientific; 0.01% v/v), dimethyl sulfoxide (DMSO; Sigma‐Aldrich; 0.1% v/v), englerin A [PhytoLab (Vesternbergsgreuth, Germany) 10 μm] for 12 h at 37°C under 5% CO_2_. The colorimetric reaction was quantified in a Multiskan FC microplate reader (Thermo Fisher Scientific) as absorbance at 405 nm with 490 nm as reference.

### Migration assay

Radius^TM^ 96‐well cell migration assay (Cell Biolabs, San Diego, CA, USA) was used for quantification of cell migration according to the manufacturer's protocol. Culture medium with different pH values was prepared by buffering with different amounts of bicarbonate (44 mm for pH 7.7, 22 mm for pH 7.4, 5.5 mm for pH 6.8, 2 mm for 6.4 mm). Experiments were done in culture medium under control conditions (nothing added), with different pH, or in the additional presence of drugs under investigation (alone or in combination) (pluronic acid (0.01% v/v), DMSO (0.1% v/v), (−)EA (PhytoLab; 10 μm), ML204 (10 μm; Tocris Bioscience, Bristol, UK) clemizole hydrochlorid (Tocris; 10 μm) for 6 h at 37°C under 5% CO_2_. Assays were performed in triplicates, the average of which was used and represents the result of one migration assay. Usually, at least three distinct and independent migration assays were carried out for each condition. Plates were photographed at 0 h and 6 or 12 h at the identical location of the initial image. Quantifications of migration ability were performed with ImageJ software.

### Transwell migration assay

Chemotactic migration was quantified using a transwell migration assay (8 mm pore size, polycarbonate membrane, Corning, Amsterdam, The Netherlands) according to the manufacturer's protocol. Cells were trypsinized and seeded into the upper chamber. The chemoattractant in the lower chamber was culture medium with or without englerin A (10 μm) and/or ML204 (10 μm). After incubation for 18 h at 37°C, cells on the top surface of the membrane were removed and cells that migrated to the bottom surface of the membrane were fixed and stained. Migrated cells in five randomly chosen fields of view were counted using ImageJ.

### RNA extraction and RT‐PCR from human medulloblastoma tissue, DAOY, ONS76 and UW228‐1 cells

Total RNA from human medulloblastoma tissue, DAOY, ONS76 and UW228 cells was extracted using RNeasy MiniKit (Qiagen, Valencia, CA, USA) according to the manufacturer's protocols. Concentration of each sample was measured with a NanoDrop 1000 Spectrophotometer (Thermo Fisher Scientific). RNA Samples with *A*_260_/*A*_280_ ratio greater than 2.0 and *A*_260_/*A*_230_ ratio in the range of ∼2.0–2.2 were used for RT‐PCR. For RT‐PCR, first‐strand cDNA was synthesized from 1 μg of total RNA with an oligo‐dT primer and the Moloney murine leukaemia virus reverse transpcriptase (Promega, Southampton, UK) according to manufacturer's protocol. For TRPC1, forward (5′‐CTTCCTCTCCATCCTCTTCC‐3′) and reverse (5′‐GTTTCTGACACCCGTAGTCC‐3′) primers were used to amplify a 273‐bp fragment from human TRPC1. For TRPC3, forward (5′‐ATGCTGCTTTTACCACTGTAG‐3′) and reverse (5′‐TGAGTTAGACTGAGTGAAGAG‐3′) primers were used to amplify a 449‐bp fragment from human TRPC3. For TRPC4, forward (5′‐TTGCCTCTGAAAGACATAACATAAG‐3′) and reverse (5′‐CTACTA ACACACATTGTTCACTGAG‐3′) primers were used to amplify a 300‐bp fragment from human TRPC4. For TRPC5, forward (5′‐TGCATTGCTCTATGCCATACGCAAG‐3′) and reverse (5′‐CCTCTGAACTAGACACACACTCCAC‐3′) primers were used to amplify a 266‐bp fragment from human TRPC5. For TRPC6, forward (5′‐CTGAGCTGTTCCAGGGCCAT‐3′) and reverse (5′‐CTCTTGATTTGGTTCCATG‐3′) primers were used to amplify a 428‐bp fragment from human TRPC6. For TRPC7, forward (5′‐GTCCGAATGCAAGGAAATCT‐3′) and reverse (5′‐TGGGTTGTATTTGGCACCTC‐3′) primers were used to amplify a 477‐bp fragment from human TRPC7. For glyceraldehyde 3‐phosphate dehydrogenase (GAPDH), forward (5′‐GGTATCGTGGAAGGACTCAT‐3′) and reverse (5′‐CCACCCTGTTGCTGTAGCCAAATTC‐3′) primers were used to amplify a 469‐bp fragment from human GAPDH. For OGR1, forward (5′‐GATGGGGAACATCACTGCAGA‐3′) and reverse (5′‐AACTGGTGGAAGCGGAAGG‐3′) primers were used to amplify a 369‐bp fragment from human OGR1. PCR reactions were optimized to 95°C for 5 min, 30 (OGR1, TRPC1, 3, 4, 6, 7), 40 (TRPC5), or 20 amplification cycles (GAPDH) at 95°C for 30 s, 56°C for 30 s, 72°C for 30 s, and a final extension of 5 min at 72°C.

### RNA extraction form primary granule cells

Experiments were performed on RNA isolated from C57BL6 wild‐type mice and from *Ogr1* knockout mice (C57BL6 genetic background). Total RNA from whole murine cerebellum or cultured murine cerebellar granule cells was extracted using RNeasy MiniKit (Qiagen) according to the manufacturer's protocols. RNA was extracted from whole murine cerebellum at distinct developmental stages (postnatal day (P)6, P8, P11, P16, P21) using two (three) distinct wild‐type (*Ogr1* knockout) litters (one to two pups from each litter). For the adult stage, two to three mice were used.

For RNA extraction from granule cells, primary granule cell cultures were established at P6 from individual litters (two distinct litters for wild‐type and three distinct litters for *Ogr1* knockout mice), and RNA was isolated at the relevant day *in vitro* (DIV) (DIV0, DIV2, DIV5, DIV10 and DIV15) from each preparation. RNA isolation from granule cells at DIV0 reflected RNA isolation on the day of granule cell culture preparation and was hence equivalent to P6. Concentration of each sample was measured by NanoDrop 1000 Spectrophotometer. RNA Samples with *A*_260_/*A*_280_ ratio greater than 2.0 and *A*_260_/*A*_230_ ratio in the range of ∼2.0–2.2 were used for quantitative real‐time RT‐PCR.

### Quantitative real‐time RT‐PCR

For reverse transcription, first‐strand cDNA was synthesized from 1 μg of total RNA with an oligo‐dT primer and the Moloney murine leukaemia virus reverse transcriptase (Promega Corp.) according to manufacturer's protocol. Quantitative real‐time RT‐PCR was performed in a total of 20 μl containing 10 μl of Taqman Universal PCR Master mix (Applied Biosystems/Thermo Fisher Scientific), 4 μl of cDNA (10 ng μl^−1^), 1 μl of 20× specific primers for Taqman Gene Expression Assays (Mm00558545_s1 for mouse OGR1, Mm00441975_m1 for mouse TRPC1, Mm00444690_m1 for mouse TRPC3, Mm00444280_m1 for mouse TRPC4, Mm00437183_m1 for mouse TRPC5, Mm01176083_m1 for mouse TRPC6, Mm00442606_m1 for mouse TRPC7, Mm99999915_g1 for mouse GAPDH, Applied Biosystems/Thermo Fisher Scientific) and 5 μl of DNase‐free water. To be used as standards for absolute cDNA quantification, corresponding amplicons were purified (QIAquick PCR Purification Kit, Qiagen) and quantified using the NanoDrop 1000 Spectrophotometer. Quantitative real‐time RT‐PCR was performed in a 96‐well clear optical reaction plate 7000 apparatus (Applied Biosystems/Thermo Fisher Scientific) and the thermal cycling conditions were: 2 min at 50°C, 10 min at 95°C, followed by 40 cycles of 15 s at 95°C and 1 min at 60°C. Results were analysed with the ABI Prism 7000 Sequence Detection System software (Applied Biosystems/Thermo Fisher Scientific). The number of gene copies was calculated using the comparative Δ*C*_t_ method described in Livak & Schmittgen (2001).

### Western blotting

Mouse cerebellum and granule cells at the indicated day were rinsed three times with ice‐cold phosphate‐buffered saline (PBS). Whole cell extracts were harvested in RIPA lysis buffer (Sigma‐Aldrich) supplemented with 100 μm sodium orthovanadate (Na_3_VO_4_), 100 μm phenylmethanesulfonyl fluoride (PMSF), and protease inhibitor cocktail (Roche, Basel, Switzerland). Protein concentrations were determined using the DC protein assay (Bio‐Rad Laboratories, Hercules, CA, USA). Fifty micrograms of protein lysate was fractionated by SDS‐PAGE and transferred onto nitrocellulose membranes (GE Healthcare, Chicago, IL, USA) using Trans‐Blot SD semi‐dry electrophoretic transfer cell (Bio‐Rad). After incubation with 5% non‐fat milk in PBS with Tween 20 (PBST) for 1 h at room temperature, membranes were washed once with PBST and incubated with antibodies against TRPC1 (a generous gift from Dr W. Schilling, Case Western Reserve University) (Goel *et al*. 2002), TRPC4 (Tsvilovskyy *et al*. 2009; Phelan *et al*. 2012), TRPC5 (Xue *et al*. 2011) or α‐tubulin (Santa Cruz Biotechnology, Dallas, TX, USA) at 4°C for 20 h. Membranes were washed with PBST three times for 10 min and incubated with horseradish peroxidase‐conjugated anti‐rabbit or anti‐mouse IgG antibodies (Jackson ImmunoResearch Laboratories, West Grove, PA, USA) for 1 h at room temperature. Membranes were then washed with PBST three times for 10 min. Proteins of interest were visualized using enhanced chemiluminescence (ECL) system (GE Healthcare) according to the manufacturer's protocols. Fluorescence intensity quantifications were performed with ImageJ software. Data were normalized to α‐tubulin levels. Western blots were carried out in duplicates and error bars indicate standard deviation.

### TRPC4 knockdown

Plasmids containing red fluorescent protein (RFP; to allow detection of successfully transfected cells) and TRPC4 shRNA or control scrambled shRNA (Origene Technologies, Rockville, MD, USA) were transfected into DAOY cells using the Amaxa Nucleofector electroporation system (Lonza, Basel, Switzerland) according to the manufacturer's instructions. Four different shTRPC4 constructs were mixed and transfected together prior to transfection; TRPC4‐targeting sequences were:

5′- GAGTGTGTCCATTCAAGTCAGAGAAGGTG -3′( TF 300814A)

5′- CTAAGGACCTACTGGATCAGACGAGAAGT -3′( TF 300814B)

5′- CCACTTGGACTGTTCATCAGGAAGCCATT -3′( TF 300814C)

5′- GTTATGAGGAACCTGGTGAAGCGATACGT -3′( TF 300814D)

Twenty‐four hours after transfection, culture medium containing 0.5 mg ml^−1^ puromycin (Invitrogen/Thermo Fisher Scientific) was added. After 7 days, the cells were trypsinized and resuspended in culture medium. Cells positive for RFP were sorted using a MoFlo^TM^ XDP high‐speed cell sorter (Beckman Coulter, Brea, CA, USA). Western blots to quantify TRPC4 knockdown were carried out as described but using 30 mg of protein lysates.

### Transfection of TRPC4β into ONS76 cells

Transient transfection was performed using Lipofectamine 2000 (Invitrogen/Thermo Fisher Scientific) according to the manufacturer's instructions. Briefly, ONS76 cells were plated in six‐well plates in DMEM containing 10% FCS without antibiotics 1 day before transfection such that they were 80–90% confluent at the time of transfection. One microgam of each plasmid and 2 μl of Lipofectamine 2000 were diluted separately in 100 μl of Opti‐MEM I Reduced Serum Medium (Invitrogen/Thermo Fisher Scientific). After 5 min of incubation at room temperature, the diluted plasmids and Lipofectamine 2000 were mixed gently and incubated for an additional 20 min at room temperature. The DNA–Lipofectamine 2000 complexes were then added to each well; cells were incubated at 37°C in a CO_2_ incubator. Transfected cells were selected by 600 μg ml^−1^ G418 for 2 weeks before experiments started.

### Organotypic cerebellar slice cultures and immunofluorescent staining

Organotypic cerebellar slice cultures were prepared as previously described (Becker *et al*. 2009). Slices were incubated with control medium or medium with 0.01% pluronic acid plus 0.1% DMSO, 10 μm (−)EA, 10 μm ML204 or 10 μm clemizole hydrochloride (CHC). Half the volume of the medium was exchanged every day. After 14 days *in vitro*, slices were fixed with 4% paraformaldehyde for 20 min at room temperature and then washed three times with PBS. Slices were then incubated with 0.05% Triton X‐100 for 20 min at room temperature and washed three times with PBST. After incubate with SuperBlock^TM^ blocking buffer (Thermo Fisher Scientific) overnight at 4°C, slices were washed once with PBST and incubated with antibodies against NeuN (Abcam, Cambridge, UK; lot no. GR270134‐1) and mouse calbindin (Synaptic Systems, Göttingen, Germany; lot number 214011/5) at 4°C overnight. Slices were washed with PBST three times for 10 min and incubated with Alexa 488‐conjugated anti‐rabbit IgG and Alex 594‐conjugated anti‐mouse IgG antibodies (Molecular Probes/Thermo Fisher Scientific) for 1 h at room temperature. After washing three times with PBST for 10 min, slices were mounted and fluorescence images were taken by using a fluorescence microscope (DMRD system, Leica, Wetzlar, Germany) with a CCD camera.

### Statistics

Statistical significance was calculated using Instat 2.03 (for Macintosh; GraphPad Software, La Jolla, CA, USA). Student's unpaired, two‐tailed *t* test was used for comparison of two means. Two‐tailed ANOVA was used for comparison of more than two means, and the Student–Newman–Keuls or Bonferroni test was used for *post hoc* analyses. Data are presented as means ± SEM unless otherwise stated, and *n* indicates number of cells used or number of repeats carried out. Asterisks indicate level of significance (^*^ *P* < 0.05, ^**^ *P* < 0.01, ^***^ *P* < 0.001, ^****^ *P* < 0.0001).

## Results

### OGR1 promotes TRPC4 expression in cerebellar granule cells

**Figure 1:**
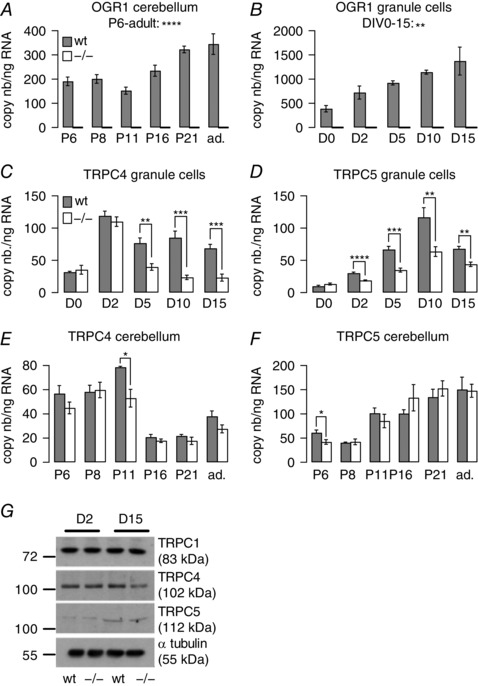
Impact of OGR1 knockout on TRPC4 and 5 expression levels in whole cerebellum and cerebellar granule cells *A*, bars reflect average OGR1 copy numbers (nb) ± SEM in RNA isolated from whole cerebellum from wild‐type (wt; grey) and *Ogr1^−/−^* (white) mice at postnatal days (P)6, 8, 11, 21 and adult (ad.). *B*, bars reflect average OGR1 copy numbers ± SEM in RNA isolated from granule cell cultures derived from wild‐type and *Ogr1^−/−^* animals at days *in vitro* (D)0, 2, 5, 10 and 15. *C–F*, bars reflect average copy numbers ± SEM of either TRPC4 (*C* and *E*) or TRPC5 (*D* and *F*) per nanogram RNA isolated from either granule cell cultures (*C* and *D*) or whole cerebellum (*E* and *F*) in wild‐type (wt; grey) and *Ogr1^−/−^* (white) tissues as a function of days *in vitro* (*C* and *D*) and postnatal age (*E* and *F*). *G*, representative Western blot depicting protein levels for TRPC1, 4, 5 and α‐tubulin (internal control) for wild‐type and *Ogr1^−/−^*‐derived granule cells at DIV2 and 15. Two‐tailed ANOVA was used to establish statistical significance over the time period indicated for *A* and *B*. For *C–F*, Student's unpaired *t* test was used to assess statistical significance between wild‐type and *Ogr1^−/−^* tissues; *n* = 4 qPCR repeats for wild‐type and 6 for *Ogr1^−/−^*‐derived RNA.

We had previously shown that differentiation of DAOY cells was accompanied by a decrease in OGR1‐dependent signalling and expression of TRPC4 levels (Huang *et al*. 2009), and we now found that TRPC4 expression levels in DAOY cells cultured at pH 7.7, when OGR1 is not active (Ludwig *et al*. 2003), were lower than in DAOY cells cultured at physiological pH, when OGR1 is partially active (Ludwig *et al*. 2003) (Supplementary Fig. 1*A*). Furthermore, we found that exposure of primary granule cells to pH 6 in Ca^2+^‐free conditions led to activation of ERK signalling (Supplementary Fig. 1*B*), mirroring our previous findings in DAOY cells (Huang *et al*. 2008). To establish whether there was also a link between OGR1 activity and TRPC4 expression in cerebellum and in particular in primary granule cells, we isolated RNA from whole cerebellum and from primary granule cell cultures derived from wild‐type and *Ogr1* knockout (*Ogr1^−/−^*) murine cerebellum at postnatal day (P)6. The day of granule cell isolation was denoted day *in vitro* 0 (DIV0), and cultures derived at this stage were a mix of granule precursor cells at various stages of differentiation and fully differentiated cells. RNA was extracted from whole cerebellum on P6, 8, 11, 16 and 21 and from adult cerebellum, and from granule cell cultures on DIV0 (i.e. cells were used directly after isolation), DIV2, 5, 10 and 15; these time points were equivalent to P6, 8, 11 16, and 21. Using quantitative PCR, the absolute number of copies for OGR1 and TRPC subunits (TRPC1, TRPC3–7) per nanogram cerebellar RNA was then established for each developmental and culturing stage in wild‐type (grey bars) and *Ogr1^−/−^* (white bars) tissue (Fig. 1). OGR1 was consistently expressed at all developmental and culturing stages in wild‐type, but not *Ogr1^−/−^*‐derived cerebellum (Fig. 1 *A*) and granule cells (Fig. 1 *B*), and its expression increased significantly with age, consistent with the report that OGR1 is most highly expressed in cerebellum (Regard *et al*. 2008).

We then compared expression levels of TRPC subunits in primary wild‐type and *Ogr1^−/−^* granule cell cultures and observed consistently significant differences only for TRPC4 and TRPC5 (Fig. 1 *C* and *D*, respectively). Both subunits showed significantly reduced levels of expression in *Ogr1^−/−^*‐derived granule cells.

We next looked for differences in TRPC4 and 5 expression levels in whole cerebellum (Fig. 1 *E* and *F*, respectively). The pattern of decreased TRPC4/5 expression observed in *Ogr1^−/−^* granule cells was not replicated. Only at P11/DIV5 were TRPC4 expression levels significantly reduced in both granule cell cultures and whole cerebellum of *Ogr1^−/−^* mice (*P* = 0.0035 and 0.0235 for granule cell cultures and whole cerebellum, respectively), but this difference did not persist at later developmental stages in whole cerebellum (Fig. 1 *E*). For TRPC5, we only found a significant reduction of expression levels at P6 between wild‐type and *Ogr1^−/−^* cerebellum (Fig. 1 *F*), which was not mirrored at DIV0 in granule cell cultures (Fig. 1 *D*).

Expression levels of TRPC1, 3, 6 and 7 in wild‐type and *Ogr1^−/−^* granule cells and whole cerebellum are shown in Supplementary Figs 2 and 3.

To see whether the observed differences in TRPC4 and 5 RNA expression translated into differences at the protein level, we carried out Western blots using protein isolated from wild‐type and *Ogr1^−/−^* granule cells at DIV2 and 15 (Fig. 1 *G* shows a representative blot). For TRPC1, we did not see any differences in protein level between wild‐type and *Ogr1^−/−^* granule cells at either DIV2 or DIV15 following quantification of protein levels (normalized TRPC1 expression at DIV2 was 1.2 ± 0.11 in wild‐type and 1.22 ± 0.1 in *Ogr1^−/−^* granule cells; at DIV15, 1.29 ± 0.11 (wild‐type) and 1.24 ± 0.08 (*Ogr1^−/−^*), consistent with our qPCR data (Supplementary Fig. 2*A*).

For TRPC4, we could not detect a difference in protein level between wild‐type and *Ogr1^−/−^* granule cells at DIV2 (normalized TRPC4 expression 1.53 ± 0.1 for wild‐type and 1.54 ± 0.1 for *Ogr1^−/−^* granule cells). However, at DIV15 TRPC4 expression levels were significantly lower in *Ogr1^−/−^* (0.9 ± 0.13) compared with wild‐type granule cells (1.62 ± 0.15; *P* = 0.0228), and also significantly lower than at DIV2 in *Ogr1^−/−^* cells (*P* = 0.0175), as expected from our qPCR results.

TRPC5 expression significantly increased between DIV2 and DIV15 in wild‐type (normalized TRPC5 expression 0.59 ± 0.11 at DIV2, 1.26 ± 0.2 at DIV15; *P* = 0.0273) and *Ogr1^−/−^* granule cells (0.66 ± 0.12 at DIV2, 1.29 ± 0.2 at DIV15; *P* = 0.0351), consistent with qPCR results, but we did not detect a difference in TRPC5 protein expression levels at DIV15 between the two preparations, contrary to qPCR results.

Hence, we only found significant differences in TRPC4 expression levels at RNA and protein level between wild‐type and *Ogr1^−/−^* granule cell cultures but not in whole cerebellum. This is a likely consequence of the culturing conditions for primary granule cells, which consist of not changing the culture medium and adding glucose to feed cells. This results in acidification of the culture medium due to metabolic activity of the cultured cells: by DIV5, the pH of the culture medium had fallen from pH 7.4 to pH 7.2, and decreased further to pH 6.8 by DIV15. Together with the increased expression of OGR1 over the same culture period, it seems likely that OGR1 is increasingly active, thus having a stronger influence on TRPC4/5 expression levels. In whole cerebellum, there is unlikely to be global acidification of the interstitial fluid, and hence OGR1 is not active to such an extent that would be detectable in terms of TRPC4/5 expression levels in whole cerebellar RNA.

### Direct activation of TRPC4‐containing channels causes small intracellular Ca^2+^ rises but does not affect proliferation or migration of granule cells

We next wanted to establish what the functional consequence of TRPC4 expression was in primary granule cells. We had previously shown that these cells and their precursors express TRPC4, and that its expression levels fall during postnatal development of the cerebellum, suggesting that TRPC4 might play a role during cerebellar development (Huang *et al*. 2007). Two obvious roles for TRPC4 would be in proliferation and migration of granule precursor cells, especially since TRP channels have been implicated in cell migration in a number of systems (Dhennin‐Duthille *et al*. 2011; Prevarskaya *et al*. 2011; Okamoto *et al*. 2012; Nesin & Tsiokas, 2014; Nielsen *et al*. 2014; Asghar *et al*. 2015; Morrone *et al*. 2016).

Generally, TRPC channels are thought to be activated in response to stimulation of receptors linking to phospholipase C, but recently (−)‐englerin A ((−)EA), a plant‐derived sesquiterpene, has been shown to be a direct agonist at TRPC4 and 5 (Akbulut *et al*. 2015).

**Figure 2:**
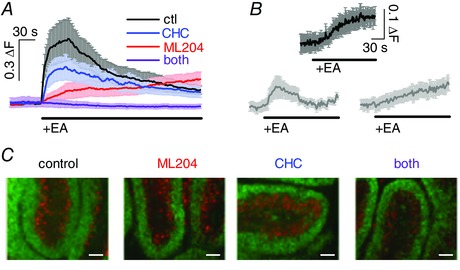
Impact of TRPC4/5 activation on normal granule cells *A*, graph showing average fluorescence signal (±SEM) in primary granule cells (DIV1/2) in response to application of 10 μm (−)englerin A (EA) under control (ctl) conditions (black), in the presence of 10 μm CHC (blue), ML204 (red), or both (purple); *n* = 17–88 granule cells . Δ*F*, fluorescence ratio (see Methods). *B*, fluorescence signals in response to application of 10 μm (−)EA in granule cells migrating through the molecular layer (top; *n* = 4), and resident in the internal granule cell layer (bottom) in acute sagittal cerebellar slices; responses were separated by response kinetics into fast (left; *n* = 9) and slow responders (right; *n* = 6). *C*, organotypic cerebellar slices after 14 days of culturing under control conditions (culture medium only), and in the presence of 10 μm ML204, 10 μm CHC, or both. Antibodies against NeuN label postmitotic granule cells (green); antibodies against calbindin label Purkinje cells (red). Scale bars are 0.1 mm.

We first examined whether or not application of 10 μm (−)EA could give rise to increases in intracellular Ca^2+^ concentration ([Ca^2+^]_i_) in fluorescence imaging experiments in primary wild‐type granule cell cultures, which would suggest the presence of Ca^2+^‐permeable TRPC4/5‐containing channels. (−)EA triggered relatively small increases in [Ca^2+^]_i_ in primary granule cells (Fig. 2 *A* black). Pharmacological inhibition of TRPC4 using 10 μm of the TRPC4 inhibitor ML204 (Miller *et al*. 2011) (Fig. 2 *A* red) or TRPC5 using 10 μm of the TRPC5 inhibitor clemizole hydrochloride (CHC) (Richter *et al*. 2014) (Fig. 2 *A* blue) partially blocked the (−)EA‐evoked Ca^2+^ signal that was only fully suppressed when both inhibitors were present (Fig. 2 *A*, purple). More primary granule cells responded to (−)EA application with intracellular Ca^2+^ signalling under conditions of TRPC4 inhibition (44.8% responders) compared with TRPC5 block (18.1% responders), suggesting that some granule cells only express (functional) TRPC5.

Small changes in [Ca^2+^]_i_ following stimulation of TRPC4/5 in granule cells were also observed in fluorescence imaging experiments subsequent to (−)EA applications in acute wild‐type cerebellar slices in the molecular layer (migrating granule precursor cells; 100% responders; Fig. 2 *B* upper panel) and the internal granule cell population (fully differentiated cells; 71.4% responders; Fig. 2 *B* lower panels). An intriguing difference between molecular and internal granule cell layer granule cells was that granule cells of the molecular layer all displayed fluorescence Ca^2+^ signals with a slow time course whereas granule cells of the internal granule cell layer were a mix of quick and slower responders.

We next tested whether activation of TRPC4/5 by (−)EA could influence granule cell proliferation rates and found that there was no difference in the presence and absence of 10 μm (−)EA (in presence of (−)EA 94.9 ± 4.7% (*n* = 3) of control proliferation rate 100 ± 2.3% (*n* = 3)). However, 10 μm AraC, an inhibitor of cell proliferation, led to a profound inhibition of granule cell proliferation (45.6 ± 5.8% of control proliferation), demonstrating that under our experimental conditions granule cells did proliferate and that lack of proliferation in response to (−)EA treatment did not reflect lack of ability to proliferate.

We then wanted to test migration of primary granule cells in response to TRPC4/5 activation. Primary granule cells do not stick to plastic surfaces, and coating of surfaces to allow granule cell attachment may interfere with granule cell motility. We therefore investigated granule precursor cell migration in organotypic cerebellar slice cultures under control conditions, and in the presence of 10 μm ML204, or CHC, or both (Fig. 2 *C*). If TRPC4/5 activation were required for migration of granule precursor cells, we would expect to see differences in migration extent and/or pattern of granule cells under the different conditions after DIV14, which would be equivalent to P21, when postnatal development of the cerebellar cortex is complete in rodents (White & Sillitoe, 2013). Postmitotic granule cells are the only cells in the cerebellar cortex that stain positively for NeuN (Weyer & Schilling, 2003), and because granule cell migration only starts after granule precursor cells exit the cell cycle (Weyer & Schilling, 2003), we used antibodies against NeuN (Fig. 2 *C*, green) to track granule cell movement. We also used antibodies against calbindin to identify Purkinje cells (Fig. 2 *C*, red) to monitor development of the cerebellar cortex in the organotypic slice culture. We found no evidence that inhibiting TRPC4/5 activity interfered with granule cell migration in the organotypic slice culture model.

Taken together, activation of TRPC4/5‐containing channels in granule cells gives rise to small increases in [Ca^2+^]_i_ but this neither affects proliferation of primary granule cells nor migration of granule precursor cells in cerebellar slice cultures.

### Direct activation of endogenous TRPC4‐containing channels causes large intracellular Ca^2+^ rises in and promotes migration of transformed granule cells

**Figure 3:**
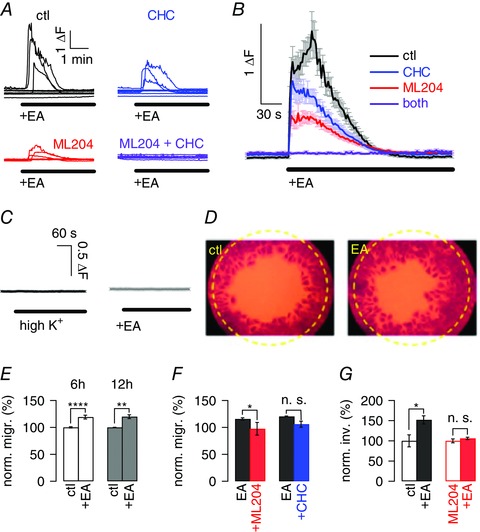
Impact of activation of TRPC4/5‐containing channels by (−)EA on transformed granule cells *A*, raw data showing 8 representative fluorescence traces in DAOY cells in response to application of 10 μm (−)EA application under control conditions (ctl; black), in the presence of 10 μm of CHC (blue), ML204 (red), or both inhibitors (purple). Δ*F*, fluorescence ratio (see Methods). *B*, average fluorescence traces (±SEM) obtained in DAOY cells exposed to (−)EA under the experimental conditions described for *A*; results include cells shown in *A*. *C*, left panel, average fluorescence signal (±SEM) in response to increasing extracellular K^+^ concentration to 12.5 mm; *n* = 38 DAOY cells. Right panel, average fluorescence signal (±SEM) in response to (−)EA application in the absence of extracellular Ca^2+^; *n* = 21 DAOY cells. Scale bar is the same for both panels. *D*, raw data showing representative migration assays; left panel shows migration under control (ctl) conditions, right panel in the presence of 10 μm (−)EA. Yellow circle indicates area for which migration was analysed. *E*, bars show average normalized (norm.) migration (migr.) (±SEM) of DAOY cells under control (ctl) conditions and in the presence of 10 μm (−)EA after 6 h (white) and 12 h (grey). Migration was normalized to average migration under control conditions for 6 or 12 h; *n* = 11 migration assays for 6 h and 3 for 12 h. *F*, bars show average migration (± SEM) of DAOY cells in the presence of 10 μm (−)EA (black), and in the additional presence of 10 μm ML204 (red), or 10 μm CHC (blue). Migration was normalized to average migration levels in the presence of (−)EA only; *n* = 4–5 migration assays. *G*, bars depicting average transwell migration as normalized invasion (norm. inv.) (±SEM) of DAOY cells under control conditions (open black) and in the presence of 10 μm (−)EA (filled black), as well as under conditions of TRPC4 block using 10 μm ML204 alone (open red) or together with 10 μm (−)EA (filled red).

We next considered the possibility that TRPC4/5‐activation may differ between normal and transformed cells and turned to DAOY cells as a model system for transformed cerebellar granule cells. Activation of TRPC4/5 using (−)EA revealed robust Ca^2+^ influx in many but not all DAOY cells tested that was much larger than that observed in granule cells. (−)EA‐mediated Ca^2+^ signals in DAOY cells were partially blocked in either CHC or ML204, and fully blocked in the combined presence of both inhibitors (Fig. 3 *A* for individual data, Fig. 3 *B* for average data). Overall, 41.5% of DAOY cells responded to (−)EA application with intracellular Ca^2+^ rises, and similar percentages of cells responded when carrying out (−)EA applications in the presence of either the TRPC4 or TRPC5 inhibitor (40.6% and 40%, respectively).

Importantly, intracellular Ca^2+^ rises in response to (−)EA application were not observed under depolarizing conditions nor in the absence of extracellular Ca^2+^ (Fig. 3 *C*). Hence, (−)EA triggered Ca^2+^ influx and not Ca^2+^ release from stores. Furthermore, the fact that depolarization of DAOY cells did not cause Ca^2+^ influx suggests that Ca^2+^ influx observed following (−)EA application is due to Ca^2+^ influx through TRPC4/5‐containing channels rather than resulting from TRPC4/5‐dependent depolarization of cells and subsequent activation of voltage‐gated channels that are Ca^2+^ permeable.

We next tested whether activation of TRPC4/5‐containing channels could affect DAOY cell survival or proliferation. DAOY cells were grown for 12 h in the absence and presence of 10 μm (−)EA in culture medium with or without 10% FCS. There was no difference in DAOY cell numbers when 10 μm (−)EA was present in either serum‐free or serum‐containing conditions (Table 2).

**Table 2:** DAOY cell numbers following exposure of cells to (−)EA for 12 h in serum‐containing and serum‐free conditions

	10% FCS	0% FCS
Control	100 ± 8.1	100 ± 1.8
+ (−)EA	98.8 ± 1.6	100.7 ± 0.3

TRPC4/5 activation also did not affect the proliferation rate of DAOY cells (Table 3). As expected, we did observe a clear reduction in proliferation in the presence of AraC, indicating that DAOY cells did indeed proliferate under our experimental conditions (Table 3). The apparently reduced effect of AraC under serum‐free conditions reflects a reduced basal proliferation rate under those conditions.

**Table 3:** Proliferation rates of DAOY cells under serum‐containing and serum‐free conditions in response to exposure to (−)EA and AraC

	10% FCS	0% FCS
Control	100 ± 2.42	100 ± 3.7
+ (−)EA	99.5 ± 2.35	99.8 ± 2.5
+ AraC	51.9 ± 3.38	63.5 ± 1.74

We then considered that TRPC4/5‐containing channels might affect DAOY cell migration and carried out migration experiments in the presence and absence of 10 μm (−)EA. Already after 6 h, there was a 19.2 ± 3.5% increase in migration in DAOY cells exposed to (−)EA (Fig. 3 *D* for raw data, Fig. 3 *E* white bars). Prolonging exposure time to 12 h did not increase the extent of DAOY cell migration (20 ± 3.5%; Fig. 3 *E* grey bars), which was significant for both 6 h (*P* < 0.0001) and 12 h (*P* < 0.01). Furthermore, the presence or absence of 10% FCS did not impact on the extent of TRPC4/5‐mediated migration; also under serum‐free conditions, (−)EA enhanced basal migration by 19 ± 5.2% (*P* < 0.05). Solvents used for (−)EA did not affect migration (100 ± 1.2% control migration, *n* = 3; 101 ± 2.9% migration in additional presence of 0.01% pluronic acid and 0.1% DMSO, *n* = 3; *P* = 0.5718).

To address whether (−)EA enhanced migration of DAOY cells by activating TRPC4/5, the extent of migration in the presence of 10 μm (−)EA, with or without 10 μm ML204 or CHC, was measured. Treatment of cells with the TRPC4 inhibitor ML204 reduced (−)EA‐mediated cell migration to basal levels (Fig. 3 *F* left bars). The TRPC5 inhibitor CHC also significantly reduced (−)EA‐mediated DAOY cell migration but to a lesser extent and less consistently so (Fig. 3 *F* right bars); this may at least in part reflect inhibition of TRPC4‐containing channels by CHC, which can occur at the concentration of CHC used here to ensure full block of TRPC5 channels (Richter *et al*. 2014). ML204 and CHC (individually and in combination) had no impact on basal migration in the absence of (−)EA (6 and 12 h migration; *P* between 0.629 and 0.832).

To confirm that TRPC4 activation did indeed enhance DAOY cell motility to the extent that invasion might be promoted, we also used a transwell migration assay that requires cells to cross a barrier, thus more closely resembling the process of migration to invade *in vivo*. (−)EA significantly enhanced transwell migration of DAOY cells (*P* < 0.027) (Fig. 3 *G* left bars). Importantly, this (−)EA‐mediated increase in transwell migration was fully blocked in the presence of 10 μm ML204 (*P* = 0.3217) (Fig. 3 *G* right bars), consistent with the idea that it was predominantly mediated through activation of TRPC4‐containing channels.

**Figure 4:**
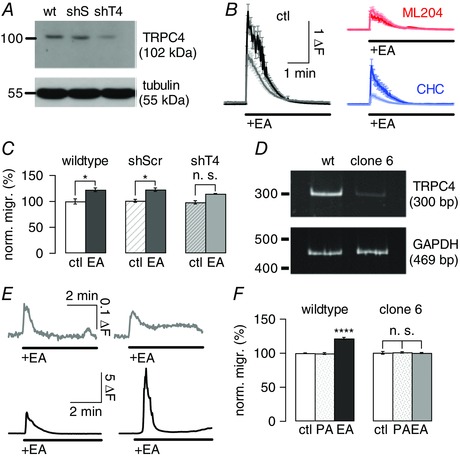
Genetic evidence for a critical role of TRPC4 in transformed granule cell migration *A*, representative Western blot showing extent of TRPC4 knock‐down in shTRPC4‐transfected DAOY cells (shT4) compared with non‐transfected wild‐type (wt) and shScramble‐transfected (shS) DAOY cells using a selective TRPC4 antibody. Tubulin was used as an internal control. *B*, graphs showing average (±SEM) fluorescence responses to 10 μm (−)EA under control conditions (left), and in the presence of 10 μm ML204 (top right) or CHC (bottom right). Black, red and blue traces depict averages obtained in non‐transfected DAOY cells, grey, light red and light blue traces depict averages obtained in shTRPC4‐transfected cells; *n* = 38‐41 cells. Δ*F*, fluorescence ratio (see Methods). *C*, bars showing mean normalized migration (norm. migr.) (±SEM) of DAOY cells in the presence of pluronic acid and DMSO (see Methods) in wild‐type, shScramble (shScr)‐ and shTRPC4 (shT4)‐transfected cells under control (ctl) conditions and in the presence of 10 μm (−)EA. Migration was normalized to mean migration levels of non‐transfected DAOY cells under control conditions; *n* = 3 repeats per condition. *D*, gel showing RT‐PCR results for cDNA generated from wild‐type DAOY cells and DAOY cell clone 6 using primers specific for human TRPC4 and GAPDH (internal control). *E*, graphs showing representative fluorescence responses to 10 μm (−)EA application in DAOY clone 6 (top two panels, grey) and in wild‐type DAOY cells (bottom two panels, black). Top scale bar applies to top panels, bottom scale bar applies to bottom panels. *F*, bars showing mean normalized migration (±SEM) of wild‐type DAOY (left bars) and DAOY clone 6 cells (right bars) under control conditions, in the presence of pluronic acid (PA) and in the presence of 10 μm (−)EA.

Since we observed full inhibition of (−)EA‐mediated migration of DAOY cells using the TRPC4 inhibitor ML204 only, we wanted to further confirm a key role for TRPC4 in DAOY cell migration and used shRNA targeted against TRPC4 to test how this impacted on DAOY migration in response to (−)EA application (Fig. 4 *A* shows one of the Western blots carried out to quantify knock‐down of TRPC4; average extent of knock‐down was 63.3 ± 19.1%).

We then assessed the fluorescence Ca^2+^ signal in response to (−)EA application in DAOY cells under control conditions and in the presence of either TRPC4 or 5 inhibitor in wild‐type cells and following knock‐down of TRPC4 (Fig. 4 *B*). There was a clear reduction in the overall fluorescence signal under control conditions upon knock‐down of TRPC4 (Fig. 4 *B* left graph; black, control, grey, following knock‐down of TRPC4). As expected, there was no change in the fluorescence Ca^2+^ signal in DAOY cells in which TRPC4 had been knocked down in the presence of 10 μm ML204 (Fig. 4 *B* top graph; red, control, light red, following knock‐down of TRPC4), demonstrating that our shTRPC4 probe indeed targeted TRPC4‐containing channels. Importantly, we observed a clear difference in the fluorescence Ca^2+^ signal upon knock‐down of TRPC4 in experiments using 10 μm CHC (Fig. 4 *B* bottom graph), showing that TRPC5 channels were not affected by our knock‐down protocol.

We then investigated whether TRPC4 knock‐down interfered with (−)EA‐mediated migration of DAOY cells in migration experiments using wild‐type, shTRPC4‐, and shScramble‐transfected DAOY cells. There was no difference in basal migration between these cells (*P* = 0.9772), but there was a significant reduction in migration in response to (−)EA in shTRPC4‐transfected compared with wild‐type or shScramble‐transfected cells: (−)EA‐dependent migration was reduced by 50% (*P* = 0.0243) (Fig. 4 *C*). Moreover, the (−)EA‐mediated increase in migration was no longer statistically significant compared with basal migration levels in these cells (*P* = 0.1216), while in wild‐type and shScramble transfected cells, the increase in migration in response to (−)EA was the same and statistically significant for both (increase by 22.3%, *P* = 0.0212, for wild‐type DAOY cells; by 22.5%, *P* = 0.0093, for shScramble‐transfected DAOY cells; Fig. 4 *C*).

To provide further evidence for a key role of TRPC4 in DAOY cell migration, we isolated individual cell clones from DAOY cell cultures. Quantitative PCR identified the DAOY clone with the lowest TRPC4 mRNA expression (DAOY clone 6; TRPC4 RNA expression reduced by 98.6 ± 1.5% compared with wild‐type DAOY cells; *P* < 0.0001) (Fig. 4 *D*). Only 2/27 DAOY clone 6 cells tested responded to (−)EA application with increases in [Ca^2+^]_i_, and both were small signals (Fig. 4 *E* top panels, grey). Intracellular Ca^2+^ signals in wild‐type DAOY cells tested on the same day and using the same solutions were on average more than 73 times larger (Fig. 4 *E* bottom panels, black). Furthermore, DAOY clone 6 cells did not show enhanced migration upon exposure to (−)EA (Fig. 4 *F*). Crucially, there was no difference in TRPC5 mRNA expression between wild‐type and clone 6 DAOY cells (100 ± 5.7% for wild‐type, 93.3 ± 10.8% for DAOY clone 6). Taken together, these findings strongly support a critical role for TRPC4 in transformed granule cell migration.

### TRPC4 and OGR1 are prominently expressed in primary human medulloblastoma tissue

**Figure 5:**
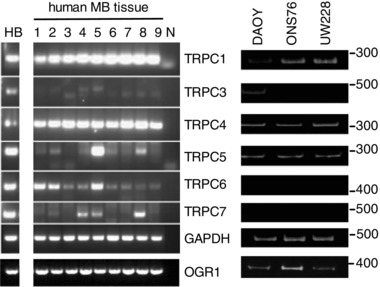
TRPC and OGR1 expression in human MB tissues and human MB cell lines RT‐PCR results using primers specific for human TRPC1, 3, 4, 5, 6 and 7 as well as human OGR1 (bottom band), using cDNA generated with RNA isolated from 9 distinct human MB tissues (see Table 1 for classification) and the human MB cell lines DAOY, UW228‐1 and ONS76. cDNA generated from healthy human brain [HB; AMS Biotechnology (Abingdon, UK) Ltd] served as positive control; N denotes negative control. GAPDH was used as internal PCR control.

We then addressed whether there was any evidence in primary MB tissue for high expression levels of TRPC4 and OGR1. Using cDNA generated from nine distinct primary human MB tissue samples, we found that OGR1 and TRPC4 were prominently expressed in all of them, as was TRPC1, whereas expression of other TRPC subunits, including notably TRPC5, was more varied between samples (Fig. 5). Similar patterns were also observed for cDNA generated from DAOY cells as well as UW228‐1 and ONS76 cells, an MB cell line considered to reflect primary MB (Zanini *et al*. 2013) (Fig. 5). These results are consistent with the idea that TRPC4 (and OGR1) might play a role in transformed cerebellar cells in general, and that our findings may also be relevant to MB subtypes other than the desmoplastic variant.

### Medulloblastoma cells overexpressing TRPC4 show large Ca^2+^ signals and migrate in response to (−)EA application

**Figure 6:**
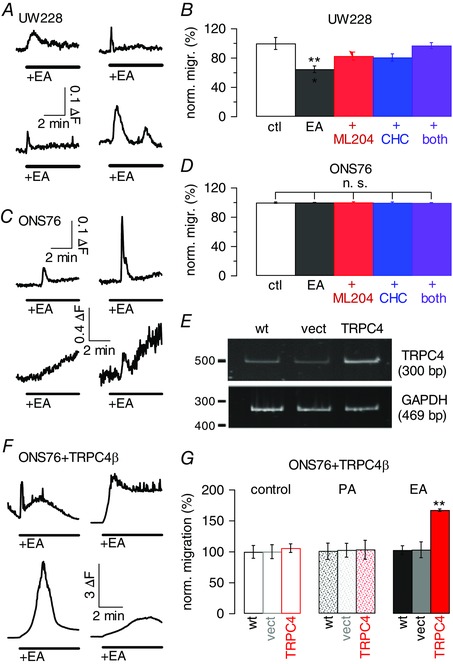
Effects of (−)EA on the MB cell lines UW228 and ONS76 *A*, representative fluorescence traces depicting changes in intracellular Ca^2+^ concentration in UW228‐1 cells upon application of 10 μm (−)EA. Scale bar applies to all traces; Δ*F*, fluorescence ratio (see Methods). *B*, bars depicting mean normalized migration (norm. migr.) under control (ctl) conditions, in the presence of 10 μm (−)EA (black), and in the additional presence of 10 μm ML204 (red), CHC (blue), or both (purple); *n* = 3 repeats. Migration was normalized to mean migration under control conditions. *C* and *D*, as *A* and *B*, respectively, but using ONS76 cells. Upper scale bar applies to upper panels, lower scale bar to lower panels. *E*, gel depicting RT‐PCR result using primers specific for human TRPC4 and GAPDH (internal control) and cDNA generated from wild‐type (wt), empty vector‐transfected (vect) and human TRPC4β‐transfected ONS76 cells. *F*, representative fluorescence traces depicting changes in intracellular Ca^2+^ concentration in ONS76 cells transfected with human TRPC4β upon application of 10 μm (−)EA. Scale bar applies to all traces. *G*, bars representing normalized migration of non‐transfected (wt), empty vector‐transfected (vect) and human TRPC4β‐transfected ONS76 cells under control conditions, in the presence of pluronic acid (PA), and in the presence of 10 μm (−)EA (red); *n* = 3 repeats per condition.

In light of the aforementioned findings, we looked for a role for TRPC4/5‐containing channels in human MB cell lines and first turned to UW228‐1 cells, which had been established from a non‐metastatic tumour of the vermis (Keles *et al*. 1995), and reflect myc‐amplified MB (Bigner *et al*. 1990). Application of 10 μm (−)EA caused small increases in [Ca^2+^]_i_ in 45.8% of cells tested (Fig. 6 *A*), similar to rises observed in granule cells. (−)EA did not promote proliferation of UW228‐1 cells, either in the presence or in the absence of serum (Table 4), and UW228‐1 cell migration was inhibited by (−)EA in a TRPC4‐ and 5‐dependent manner (Fig. 6 *B*). The inhibitors, alone or in combination, did not affect basal migration levels of UW228‐1 cells (103.8 ± 1.2% of basal migration in presence of ML204, 101.6 ± 3.6% in presence of CHC, 100.6 ± 5.4% in presence of both; data not shown). Furthermore, exposure of UW228‐1 cells to (−)EA under serum‐free conditions resulted in the same level of inhibition of migration as in the presence of serum (35.3 ± 4.5% inhibition in 10% FCS; 31.3 ± 8.6% inhibition in 0% FCS; *n* = 3).

**Table 4:** Lack of impact of TRPC4/5 activation on UW228‐1 and ONS76 cell proliferation

	Control	+ 10 μm (−)EA	+ 10 μm AraC
UW228‐1 10% FCS	100 ± 2.4%	96.5 ± 3.1%	61.9 ± 4%
UW228‐1 0% FCS	100 ± 3%	96.7 ± 3.3%	70.7 ± 4.2%
ONS76 10% FCS	100 ± 3.5%	101.6 ± 1.5%	46.7 ± 2.14%
ONS76 0% FCS	100 ± 3.3%	100.3 ± 4.6%	66.9 ± 2.7%

We then looked at impact of TRPC4/5 activation on proliferation and migration of ONS76 cells. These cells were derived from MB of the vermis, likely reflecting classical MB (Coluccia *et al*. 2016) that had disseminated within the brain (Yamada *et al*. 1989). Application of (−)EA to ONS76 cells led to small increases in [Ca^2+^]_i_ (Fig. 6 *C*), similar to those observed in primary granule cells and UW228‐1 cells. (−)EA did not promote proliferation of ONS76 cells in the presence or absence of serum (Table 4), and it also did not affect ONS76 cell migration (Fig. 6 *D*), nor did inhibition of TRPC4 and 5 individually or in combination in the absence of (−)EA affect basal cell migration (100.2 ± 1.3 of basal migration in ML204; 100 ± 0.5% in CHC; 99.8 ± 0.8% in both).

Since application of (−)EA had no effect on ONS76 migration and only produced small [Ca^2+^]_i_ rises, we addressed whether overexpression of TRPC4 in these cells might yield larger Ca^2+^ rises and promote cell migration. Transfection of human TRPC4β into ONS76 cells was confirmed by PCR (Fig. 6 *E*) and resulted in large [Ca^2+^]_i_ rises upon (−)EA application that were, on average, more than 61 times larger than those observed in ONS276 cells transfected with empty vector or wild‐type ONS76 cells (Fig. 6 *F*). It did not affect TRPC5 expression levels (100 ± 10.4% in wild‐type, 93.4 ± 1.7% in vector‐ and 94 ± 0.2% in TRPC4β‐transfected ONS76 cells). Importantly, TRPC4β‐transfected ONS76 cells displayed increased migration following exposure to (−)EA, unlike wild‐type or empty vector‐transfected ONS76 cells (Fig. 6 *G*).

### OGR1 stimulation causes Ca^2+^ influx through TRPC4‐containing channels but does not promote transformed granule cell migration

TRPC4 channels can be activated in response to stimulation of G_q_‐coupled GPCRs. We have previously shown that, in DAOY cells, activation of OGR1 causes the opening of TRP‐like, Ca^2+^‐permeable channels (Huang *et al*. 2008), and that differentiation of DAOY cells resulted in altered signalling through OGR1, including loss of Ca^2+^ influx in response to OGR1 activation (Huang *et al*. 2009). Furthermore, OGR1 is proton‐activated and TRPC4 is proton‐potentiated, and it seems reasonable to suggest that activation of a proton‐sensing GPCR would lead to activation of an ion channel that is proton‐potentiated, rather than inhibited by the acidic conditions. We therefore wanted to explore whether or not there might be a more immediate impact of OGR1 activation on TRPC4, in addition to TRPC4 expression levels being affected by OGR1.

**Figure 7:**
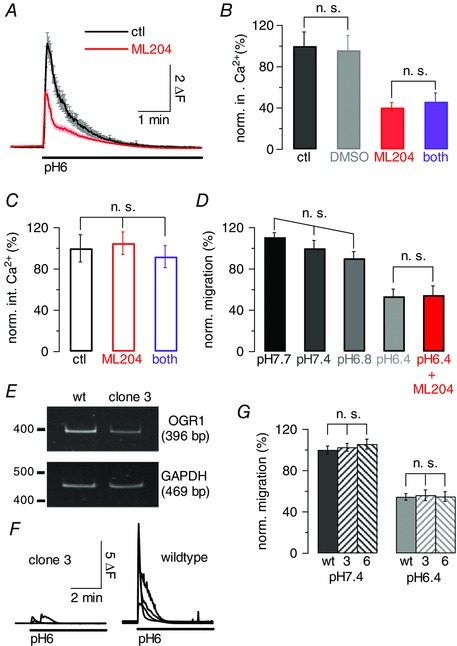
Activation of OGR1 results in opening of TRPC4‐containing channels but does not promote migration of DAOY cells *A*, graph showing average fluorescence signal (±SEM) in DAOY cells in response to a drop in extracellular pH from pH 7.35 to pH 6 to activate OGR1 under control conditions (black) and in the presence of 10 μm ML204; Δ*F*, fluorescence ratio (see Methods). *B*, normalized (norm.) average fluorescence integrals (int.) (±SEM), in response to drop in extracellular pH as for *A* under control conditions (black), in the presence of 0.2% DMSO, or 10 μm ML204 (red), or 10 μm ML204 + 10 μm CHC (purple). Integrals were normalized to average fluorescence integrals under control conditions; same cells as in *A* for control and ML204 results. Only non‐significant (n.s.) differences are indicated. *C*, as *B*, but experiments carried out in the absence of extracellular Ca^2+^, to control for impact of ML204 and CHC on Ca^2+^ release from stores. *D*, bar graph showing mean normalized migration (±SEM) of DAOY cells under increasingly acidic extracellular pH conditions; last bar (red) reflects exposure of cells to pH 6.4 and 10 μm ML204. Migration was normalized to mean migration levels at pH 7.4; 3 repeats per condition. Only non‐significant differences are indicated. *E*, gel showing RT‐PCR results for cDNA generated from wild‐type DAOY and DAOY clone 3 cells using primers specific for human OGR and GAPDH (internal control). *F*, each panel shows 4 representative fluorescence traces obtained from wild‐type DAOY (right) and DAOY clone 3 (left) in response to a drop in extracellular pH from pH 7.35 to pH 6; experiments were performed in the absence of extracellular Ca^2+^ to monitor OGR1 activation only. Scale bar applies to both panels. *G*, bars reflecting average normalized migration of wild‐type (wt) DAOY, DAOY clone 3 (3) and DAOY clone 6 (6) cells at extracellular pH 7.4 (left bars) and pH 6.4 (right bars); 3 repeats per condition. Only non‐significant differences are depicted.

OGR1 activation in response to a drop in extracellular pH from pH 7.35 to pH 6 under control conditions and in the additional presence of 10 μm ML204 resulted in significant reduction of the fluorescence Ca^2+^ signal under conditions of TRPC4 block (*P* < 0.0001) (Fig. 7 *A*); additional inhibition of TRPC5 had no further impact (Fig. 7 *B*).

Importantly, repetition of experiments in the absence of extracellular Ca^2+^ did not result in a decrease of the fluorescence signal in the presence of TRPC4 (and 5) inhibitor (Fig. 7 *C*), demonstrating that OGR1‐mediated Ca^2+^ release was not affected by the drugs.

Finally, we wanted to establish whether TRPC4 activation via OGR1 could promote migration of DAOY cells. Migration assays were carried out under different extracellular pH conditions, from pH 7.7 to pH 6.4. Extracellular acidosis inhibited migration of DAOY cells (Fig. 7 *D*); this was significant for pH 6.4 (*P* = 0.0004) and independent of pharmacological TRPC4 block (Fig. 7 *D* red), unlike UW228‐1 cell migration inhibition following (−)EA application. Acidification of the extracellular medium will affect most if not all membrane proteins. To establish whether there was a correlation between OGR1 and TRPC4 expression and acidosis‐mediated inhibition of DAOY cell migration, we used the DAOY cell clone with the lowest OGR1 mRNA expression (DAOY clone 3; Fig. 7 *E*). Decreases in extracellular pH from pH 7.35 to pH 6 resulted in a more than 6 times smaller average increase in [Ca^2+^]_i_ in clone 3 compared with wild‐type DAOY cells (Fig. 7 *F*), demonstrating that DAOY clone 3 indeed expressed lower levels of functional OGR1. Crucially, basal migration was affected to the same extent by a drop in extracellular pH in wild‐type, clone 3 and clone 6 DAOY cells, suggesting that the acidosis‐mediated reduction in migration was independent of OGR1 and TRPC4.

## Discussion

In this study, we investigated a correlation between OGR1 and TRPC channel expression and functional consequences of OGR1 and TRPC4 activation in the context of normal and transformed cerebellar cells that are commonly used as model systems for studying MB. We focused on TRPC4 because of its high expression rate in primary MB tissue.

We previously showed that activation of OGR1 promoted phosphorylation and hence stimulation of the ERK pathway in DAOY cells (Huang *et al*. 2008), and we have now found that this is also true for non‐transformed granule (precursor) cells. Furthermore, we have found that OGR1 knockout affects TRPC4 expression in granule cells, and that conditions favouring OGR1 activity promote TRPC4 expression in DAOY cells. Whilst our results do not allow us to conclude that OGR1 promotes TRPC4 expression via activation of the ERK signalling pathway, this remains a possibility. ERK signalling has been shown to enhance MB cell survival, migration and invasion as well as to promote MB cell apoptosis and inhibit MB formation (Calabrese *et al*. 2003; Sturla *et al*. 2005; Włodarski *et al*. 2006; Bhoopathi *et al*. 2008; Salvatore Zavarella *et al*. 2009; Yuan *et al*. 2010; Jóźwiak *et al*. 2011). The discrepancy reflects distinct preparations or cell types used and differences in receptors activated to stimulate ERK activation, and it is therefore likely that the impact of ERK activation has to be seen in context with stimulation of other signalling pathways. ERK activation was also shown to enhance granule precursor cell differentiation (Fogarty *et al*. 2007) without affecting proliferation of these cells (Guldal *et al*. 2012). It will be important to establish in the future how OGR1 activation links to ERK phosphorylation, if TPRC4 is a target for the ERK signalling pathway, and what (other) effects OGR1‐mediated ERK activation has on granule precursor cells and their transformed counterparts.

When we investigated the physiological consequences of TRPC4 activation, we found that only DAOY cells showed prominent [Ca^2+^]_i_ rises and enhanced migration following application of (−)EA. Granule cells (in culture or in acute slices), ONS76 and UW228‐1 cells did not respond to (−)EA application with large increases in [Ca^2+^]_i_, nor did they show increased motility in the presence of (−)EA. In fact, application of (−)EA caused inhibition of UW228‐1 cell migration, which appeared to depend on activation of both TRPC4 and 5. How TRPC4/5 activation interfered with UW228‐1 cell migration is unclear but may involve TRPC4/5‐dependent depolarization of the cell membrane, which has been shown to inhibit migration in some cells (Schwab *et al*. 2012; Pappalardo *et al*. 2016).

Why activation of endogenous TRPC4/5 channels did not give rise to large Ca^2+^ signals and enhanced migration in granule, ONS76 and UW228‐1 cells is unclear but may reflect the fact that TRPC1 and 4/5 can form heteromultimers (Freichel *et al*. 2014) that have reduced Ca^2+^ permeability compared with TRPC1‐devoid channels (Storch *et al*. 2012). Hence, the smaller Ca^2+^ rise in granule, ONS76 and UW228‐1 cells may be the result of lower expression of only TRPC4/5‐containing channels, and/or of expression of heteromultimers containing TRPC1. This is in agreement with our finding that transfection of TRPC4 into ONS76 cells resulted in large Ca^2+^ signals and enhanced mobility of these cells in response to (−)EA, as overexpression of TRPC4 likely results in increased expression of TRPC4 monomeric channels. In this context it is noteworthy that TRPC1, like TRPC4, was prominently present in all primary MB samples. Given that TRPC1 subunits can modulate the Ca^2+^ permeability of TRPC4/5‐containing channels, whether or not they promote motility of MB cells may therefore depend on the subunit composition of these channels, which may differ between different MB types and stages.

We also provide evidence that OGR1 can couple to TRPC4 on two different levels: OGR1 expression enhances TRPC4 expression, and OGR1 activation leads to opening of TRPC4 channels. This finding is intriguing for several reasons. OGR1 is a proton‐activated GPCR, and TRPC4 (and 5) subunits can form channels whose function is potentiated by extracellular protons (Semtner *et al*. 2007). Hence, OGR1 appears to increase signalling pathways that are enhanced under conditions of extracellular acidosis, which may reflect a positive feedback system to promote proton signalling under these conditions.

Furthermore, TRPC4 cell surface expression has been shown to depend on its PDZ‐interacting domain binding to the scaffold protein EBP50 following phosphorylation of ezrin (Mery *et al*. 2002), a protein located between inner membrane leaflet and cytoskeleton that plays a key role in a range of distinct cellular processes including cell motility (Neisch & Fehon, 2011). Intriguingly, ezrin is overexpressed in a number of distinct cancers (Clucas & Valderrama, 2014), where it correlates with invasiveness, cell migration and metastasis (Moleirinho *et al*. 2013), and in the context of MB has been identified as a risk factor (Park *et al*. 2003; Osawa *et al*. 2009).

Additionally, in granule cells, OGR1 is under inhibitory control of CaSR, which limits signalling through OGR1 under physiological conditions (Wei *et al*. 2015). DAOY cells, transformed granule cells, also express CaSR, but it does not appear to regulate OGR1 signalling (Wei *et al*. 2015). OGR1 is already active at physiological pH (Ludwig *et al*. 2003). Hence, OGR1 can stimulate TRPC4 expression in DAOY cells, meaning that TRPC4‐containing channels are available to promote migration in response to an appropriate stimulus. In granule cells, however, OGR1 signalling and hence TRPC4 expression is limited under physiological conditions, and activation of TRPC4 does not cause large Ca^2+^ rises or migration of granule cells. This brake on TRPC4 expression may help ensure guided migration of granule cells from the external to the internal granule cell layer in a cue‐dependent manner.

There is increasing evidence of TRPC4/5‐containing channels playing a role in a variety of cancers (Gaunt *et al*. 2016). TRPC5 channels have been shown to promote drug resistance in breast cancer cells (Ma *et al*. 2012), and TRPC5‐containing channels can be transported via extracellular vesicles to other cells, thereby essentially transmitting drug resistance to the recipient cells (Ma *et al*. 2014). Furthermore, activation of TRPC4/5‐containing channels in renal cancer cells leads to their Ca^2+^‐dependent cell death (Akbulut *et al*. 2015) and inhibits cancer cell proliferation (Carson *et al*. 2015). In contrast, activation of TRPC4‐containing channels in ovarian carcinoma cells enhances their proliferation (Zeng *et al*. 2013), and it has been suggested that the opposite effects of TRPC4 activation in different cancer cell lines may reflect the fact that different levels of intracellular Ca^2+^ rises may cause different effects in cells (Gaunt *et al*. 2016). Our results suggest that TRPC4/5 activation can promote motility of certain types of MB. Enhanced motility is required for transformed cells to engage in invasion and metastasis. DAOY cells were derived from a metastatic desmoplastic MB sample, and hence the cell of origin is likely to be a granule precursor cell. However, DAOY cells do not experience inhibition of OGR1 signalling by CaSR, and thus inhibition of TRPC4 expression, unlike granule (precursor) cells. It is therefore tempting to speculate that dysregulated TRPC4 activity contributes to the progression of desmoplastic MB.

Our results provide a first insight into roles for OGR1 and TRPC4‐containing channels in MB cells. Future experiments need to address how knockout of TRPC4 impacts on the ability of DAOY, UW228‐1 and ONS76 cells to form solid tumours, as well as their invasion and dissemination.

## Additional information

### Competing interests

The authors declare that they have no competing interest with the contents of this article.

### Author contributions

W.C.W. carried out experiments for which data are shown in all figures including Supplementary material, analysed data and contributed to writing the paper; W.C.H. carried out experiments shown in Fig. 5 (human MB tissue) and Supplementary Fig. 1*B* and contributed to writing the paper; Y.P.L. carried out the transwell migration assay (Fig. 3) and contributed to writing the paper; E.B.E.B. helped with organotypic slice culture experiments and contributed to writing the paper; O.A. provided human MB tissue, classified MB samples and revised the manuscript critically; V.F. provided TRPC antibodies and human TRPC4β clone, contributed to writing and critically revised the paper; D.C. and G.C. provided ONS76 and UW228‐1 cells; M.D.G. analysed data, carried out isolation of DAOY cell clones, supervised the research and wrote the paper. All authors approved the final version of the manuscript and agree to be accountable for all aspects of the work in ensuring that questions related to the accuracy or integrity of any part of the work are appropriately investigated and resolved. All persons designated as authors qualify for authorship, and all those who qualify for authorship are listed.

### Funding

This work was supported by BBSRC (BB/1008748/1) and Sonderforschungsbereich (SFB/TR 152).

## Supporting information

Disclaimer: Supporting information has been peer‐reviewed but not copyedited.

**Supplementary Figure 1**. Preliminary data leading to study.

**Supplementary Figure 2**. TRPC1, 3, 6 and 7 subunit expression patterns in wildtype and *Ogr1^−/−^* cerebellar granule cells throughout their culturing period.

**Supplementary Figure 3**. TRPC1, 3, 6 and 7 subunit expression patterns in wildtype and *Ogr1^−/−^* cerebellum throughout postnatal development.
