# Supplementary material for: Functional expression of calcium‐permeable canonical transient receptor potential 4‐containing channels promotes migration of medulloblastoma cells
Source: J Physiol. 2017 Jul 20;595(16):5525–44. doi: 10.1113/JP274659 (PMC5556167; doi:10.1113/JP274659)
Supplement: Supplementary file 1 — Supplementary Figure 1. Preliminary data leading to study. [file TJP-595-5525-s001.pdf]

**Supplementary Figure 1: Preliminary data leading to study**

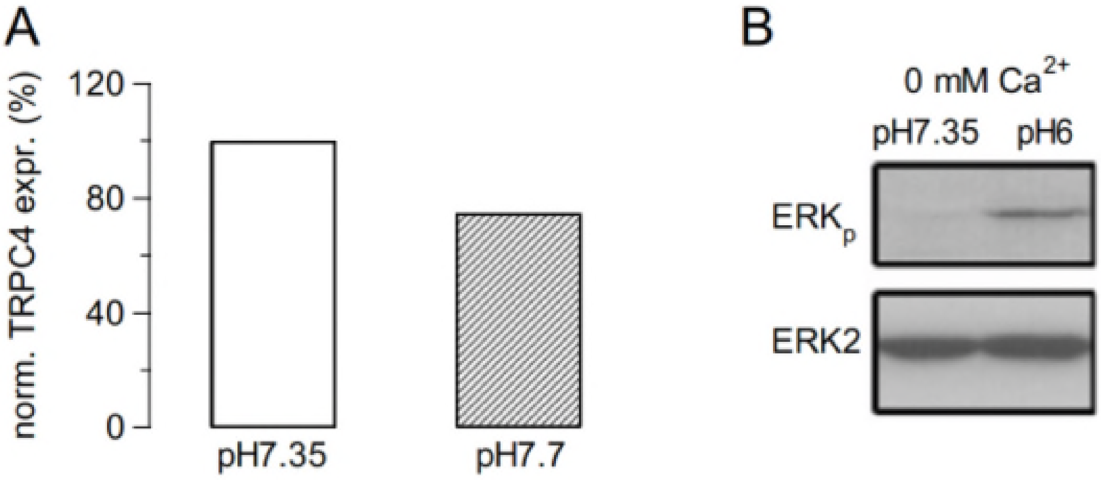


A, qPCR results showing that TRPC4 expression levels, normalised (norm.) to average TRPC4 expression (expr.) at pH7.35, are higher when DAOY cells are cultured at pH7.35 than at pH7.7; n=2 repeats for each pH condition, values shown represent averages of the two repeats.

B, Western Blot showing extent of phosphorylation of ERK protein ($ERK_p$) in response to control conditions (in absence of extracellular $Ca^{2+}$ at pH7.35) and under test conditions (in absence of extracellular $Ca^{2+}$ at pH6); 10 min exposure time for both conditions. Experiments were performed in primary granule cells at DIV1. ERK2 protein levels served as internal control.
