# Supplementary material for: Functional expression of calcium‐permeable canonical transient receptor potential 4‐containing channels promotes migration of medulloblastoma cells
Source: J Physiol. 2017 Jul 20;595(16):5525–44. doi: 10.1113/JP274659 (PMC5556167; doi:10.1113/JP274659)
Supplement: Supplementary file 2 — Supplementary Figure 2. TRPC1, 3, 6 and 7 subunit expression patterns in wildtype and Ogr1−/− cerebellar granule cells throughout their culturing period. [file TJP-595-5525-s002.pdf]

**Supplementary Figure 2: TRPC1, 3, 6 and 7 subunit expression patterns in wildtype and *Ogr1*$^{-/-}$ cerebellar granule cells throughout their culturing period.**

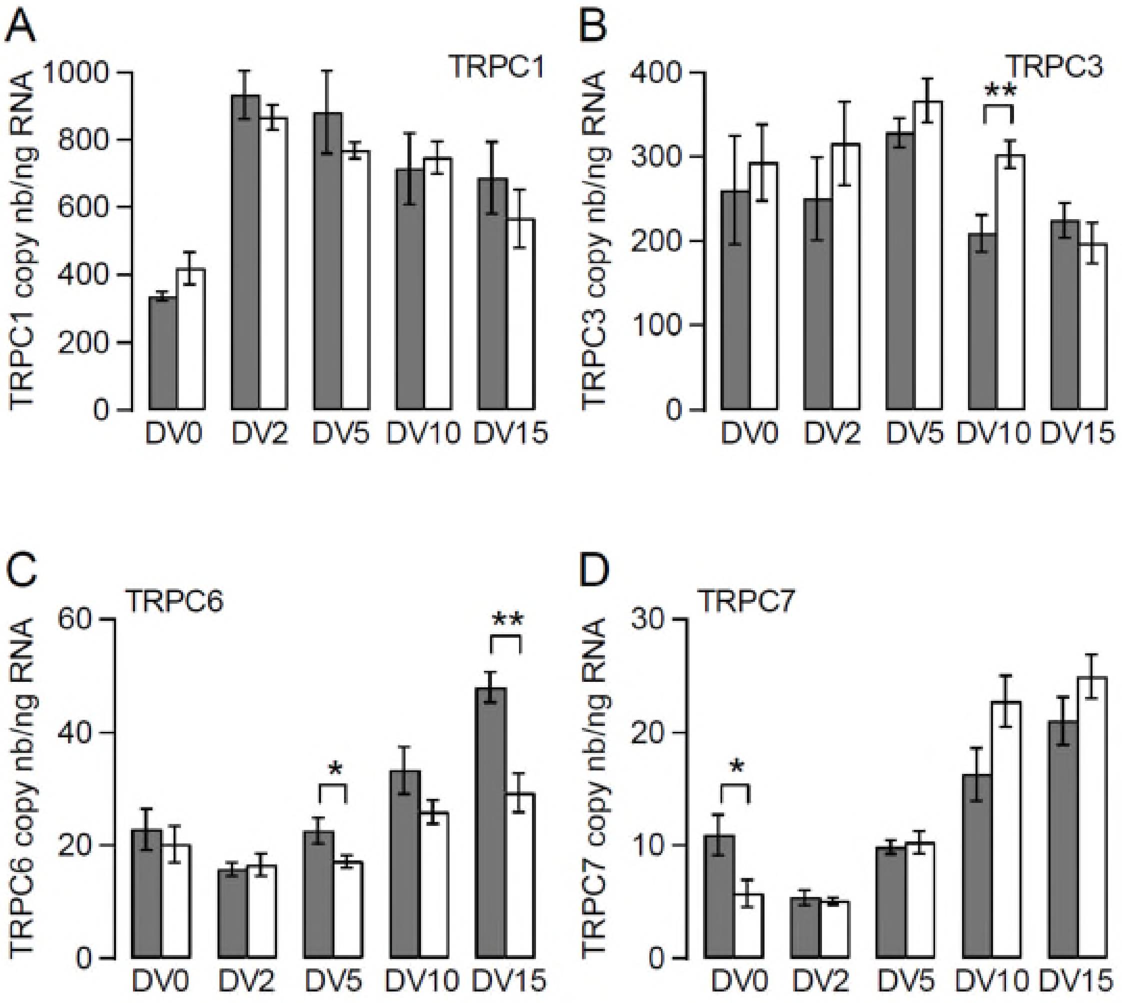


Comparison of absolute levels of TRPC1, 3, 6 and 5 subunit expression in RNA isolated from wildtype (grey) and *Ogr1*$^{-/-}$ (white) granule cells at different culturing stages. RNA was isolated on day of granule cell preparation (=day *in vitro* (DV)0), after 2 (DV2), 5 (DV5), 10 (DV10) or 15 (DV15) days in culture. Each panel depicts time-dependent expression patterns for a given TRPC subunit throughout the culturing period (DV0–DV15): TRPC1 (**A**), TRPC3 (**B**), TRPC6 (**C**), TRPC7 (**D**). Error bars are SEM; n=4 qPCR repeats for wildtype and 6 for *Ogr1*$^{-/-}$ derived RNA.
