# Supplementary material for: Functional expression of calcium‐permeable canonical transient receptor potential 4‐containing channels promotes migration of medulloblastoma cells
Source: J Physiol. 2017 Jul 20;595(16):5525–44. doi: 10.1113/JP274659 (PMC5556167; doi:10.1113/JP274659)
Supplement: Supplementary file 3 — Supplementary Figure 3. TRPC1, 3, 6 and 7 subunit expression patterns in wildtype and Ogr1−/− cerebellum throughout postnatal development. [file TJP-595-5525-s003.pdf]

Supplementary Figure 3: TRPC1, 3, 6 and 7 subunit expression patterns in wildtype and *Ogr1*$^{-/-}$ cerebellum throughout postnatal development.

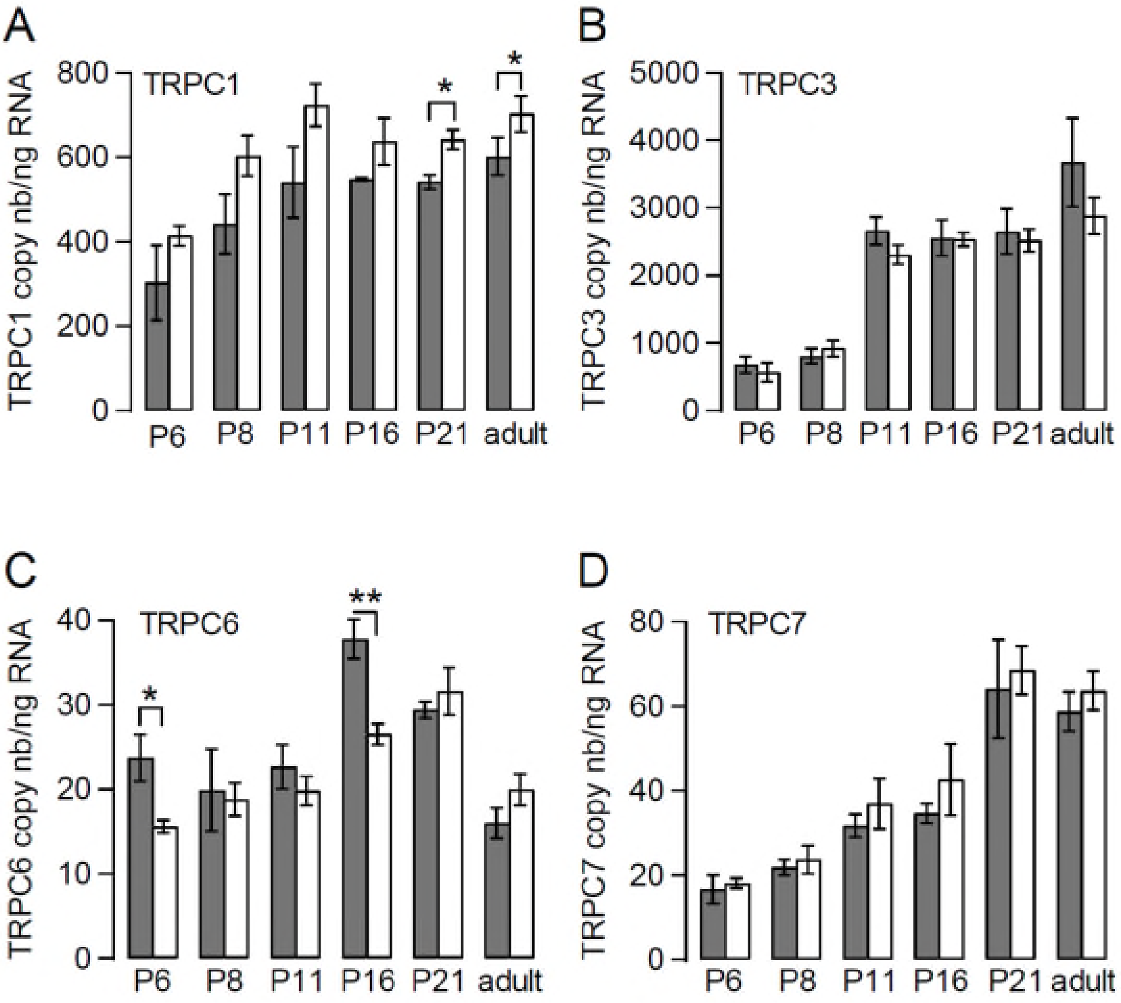

Comparison of absolute levels of TRPC1, 3, 6 and 5 subunit expression in RNA isolated from wildtype (grey) and *Ogr1*$^{-/-}$ (white) whole cerebellum at different postnatal ages. RNA was isolated on day of granule cell preparation (=postnatal day (P)6, P8, P11, P16, P21 and from adult cerebellum. Each panel depicts time-dependent expression patterns for a given TRPC subunit throughout its postnatal development (P6 - adult): TRPC1 (**A**), TRPC3 (**B**), TRPC6 (**C**), TRPC7 (**D**). Error bars are SEM; n=4 qPCR repeats for wildtype and 6 for *Ogr1*$^{-/-}$ derived RNA.
